# Early Passive Leg Movement Prevents Against the Development of Heart Failure With Preserved Ejection Fraction in Rats

**DOI:** 10.3389/fcvm.2021.655009

**Published:** 2021-04-21

**Authors:** Jian Liu, Xi-xin Ji, Yang Fu, Wen-chao Zhang, Hui-fang Ji, Jian-wei Liu, Xiao-shu Cheng, Yi-Fei Dong

**Affiliations:** ^1^Department of Cardiovascular Medicine, The Second Affiliated Hospital of Nanchang University, Nanchang, China; ^2^The First People's Hospital of Yongkang, Yongkang, China

**Keywords:** heart failure with perserved ejection fraction, pre-clinical diastolic dysfunction, early passive leg movement, cardiac fibrosis, vascular fibrosis

## Abstract

Exercising was reported by several studies to bring great benefits to heart failure with preserved ejection fraction (HFpEF), which reduced the hospitalization and the mortality of heart failure. However, the underlying mechanism of exercising on HFpEF remains unclear. In the present study, we designed and constructed a device that can perform early passive leg movement (ePLM) in rats and further observed whether treatment of ePLM exerts protective effects on HFpEF of rats. Rats were fed with high salt feed to establish an animal model of pre-clinical diastolic dysfunction (PDD), which would eventually develop into HFpEF, and then treated rats with ePLM. We conducted several experiments to evaluate the conditions of heart and blood vessel. The results show that diastolic functions of heart and blood vessel in rats were significantly improved by treatment of ePLM. We also found that pathological injuries of heart and blood vessel were ameliorated after treatment of ePLM. Moreover, treatment of ePLM decreased the protein levels of Collagen type I, Collagen type III, MMP2, and MMP9 in heart and blood vessel, indicating that cardiac and vascular fibrosis were reduced apparently by treatment of ePLM. Further investigation suggested that treatment of ePLM probably inhibit the activation of TGF-β1/Smad3 signaling pathway as well as promote the activation of Akt/eNOS signaling pathway in high salt diet induced HFpEF. In conclusion, treatment of ePLM alleviated high salt diet induced HFpEF by inhibiting fibrosis *via* suppressing TGF-β1/Smad3 signaling pathway as well as activating Akt/eNOS signaling pathway, implicating treatment of ePLM as a promising novel non-pharmacological approach for HFpEF.

## Introduction

Heart failure with preserved ejection fraction (HFpEF), occupied about 50% of heart failure population, was defined as a peculiar heart failure, which displays diastolic dysfunction but maintains a normal ejection fraction (1, 2). HFpEF is a chronic disease and deteriorates gradually with aging (3). The pathological changes in tissues of heart and blood vessel serve as the major mechanisms to the disease (4, 5). Several studies have reported that HFpEF resulted in a large number of morbidity and mortality (6). While there was no effective therapeutic methods to improve the prognosis of HFpEF to date (7). Recently, a novel study named PARALLAX trial show that sacubitril/valsartan can ameliorate the outcome of HFpEF, but it needs more investigations to confirm the theory (8). According to these situations, it is necessary to find new therapeutic methods for HFpEF.

Pre-clinical diastolic dysfunction (PDD) is the early stage of cardiac dysfunction, which do not have typical heart failure symptoms and signs, and can only be diagnosed by some auxiliary examinations like echocardiography (9). PDD show a high morbidity of about 27.4% among normal population (10). Several studies suggested that PDD mainly develop into HFpEF (11). Therefore, treatment of PDD might prevent against the development of HFpEF.

Researchers of TOPCAT trial reported that the HFpEF patients who took more exercises had a better prognosis compared to the HFpEF patients who just took little exercises, as evidenced by the facts that the adverse incident risk, hospitalization rate of heart failure, and all-cause mortality rate were lower in the HFpEF patients who took more exercises than the HFpEF patients who just took little exercises (12). Several studies confirmed the conclusion that the HFpEF patients benefit a lot from exercise training (13). These results revealed that exercise training can act as a novel non-pharmacological approach for HFpEF. Instrumental assisted passive movements serve as a simple and effective exercise training (14). Thus, we designed and constructed a device of early passive leg movement (ePLM) to treat rats with PDD and evaluated whether treatment of ePLM can improve the prognosis of HFpEF in rats, and further explored the potential mechanisms.

## Materials and Methods

### Materials

Male Dahl/Salt Sensitive (Dahl/SS) rats (6 weeks old, 150–200 g) were purchased from Beijing Charles River Laboratory Animal Co., Ltd. (Beijing, China). High salt feed (8% NaCl) and Low salt feed (0.3% NaCl) that used to constructed the animal model were obtained from Beijing keao xieli Feed Co., Ltd. (Beijing, China). Passive leg movement device on rats, which was applied for a patent (No. ZL201821187645.0, China), was designed and constructed for treating rats. The working principle of this device is that the rats were anesthetized by continuous inhaling isoflurane at a supine position in the device and then turn on the switch to make an early passive leg movement (ePLM) by the device (Supplementary Figure 1). The antibodies applied to the present study are as follows: Collagen type I antibody (Cat. No. 14695-1-AP; Proteintech), Collagen type III antibody (Cat. No. 22734-1-AP; Proteintech), MMP2 antibody (Cat. No. ab97779; Abcam), MMP9 antibody (Cat. No. ab58803; Abcam), TGF-β1 antibody (Cat. No. 21898-1-AP; Proteintech), p-Smad3 antibody (Cat. No. CST-9510S; Cell Signaling Technology), Smad3 antibody (Cat. No. CST-5678S; Cell Signaling Technology), SOD-1 antibody (Cat. No. ab51254; Abcam), p-Akt antibody (Cat. No. ab38449; Abcam), Akt antibody (Cat. No. ab8805; Abcam), p-eNOS antibody (Cat. No. CST-9570; Cell Signaling Technology), eNOS antibody (Cat. No. CST-32027; Cell Signaling Technology), GAPDH antibody (Cat. No. ab8245; Abcam), Tubulin antibody (Cat. No. ab7291; Abcam). The enzyme-linked immunosorbent assay (ELISA) kits for measurement of NT-ProBNP in serum of rats was purchased from Elabscience Biotechnology Co., Ltd. (Wuhan, China). All other reagents used in this study are common and can be acquired easily.

### Animal Model

The treatments in rats were performed in accordance with the guidelines of the Animal Care and Use Committee of the Second Affiliated Hospital of Nanchang University (China). In the present study, a total number of 48 male Dahl/SS rats were used for experimental operations. Rats were group-housed in the Animal Center of Jiangxi Medicine of Nanchang University within a 12-h light/dark cycle, with ambient temperature maintaining at 23 ± 2°C and relative humidity at 50 ± 10%.

In order to adapt the housing conditions, rats first were fed with Low salt feed (0.3% NaCl) for 7 days before any experimental operations. Then the rats were roughly and randomly divided into two groups and given different treatments: (1) Normal group (NS, *n* = 12): The rats in this group were fed with Low salt feed (0.3% NaCl) consistently until sacrificed. (2) High Salt group (HS, *n* = 36): The rats in this group were fed with High salt feed (8% NaCl). After 5 weeks feeding, all rats were gathered together for measurements of echocardiography and blood pressure analysis to evaluate the cardiac function, and we successfully constructed an animal model of PDD in High Salt group (Table 1). Next, the rats of High Salt group were further divided into three groups randomly with different treatments for 8 weeks: (1) High Salt group (HS, *n* = 12): Treatments on rats were as before. (2) Early Passive Leg Movement group (HS+ePLM, *n* = 12): Apart from feeding with High salt feed (8% NaCl), the rats were given treatment of ePLM with a passive leg movement device in the condition that rats were anesthetized *via* continuous inhaling isoflurane, with exercise time of 5 days per week, 20 min per day, and 120 revolutions per minute. (3) Isoflurane group (HS+ISO): The treatments of this group were approximately same to the ePLM group except not giving treatment of ePLM. Rats were anesthetized by continuous inhaling isoflurane at a supine position to eliminate the influence of posture and isoflurane and reduce the variables. With 8 weeks treatments, we saw a typical HFpEF in rats of HS group *via* various measurements like echocardiography.

**Table 1:** Echocardiographic data and blood pressure of rats were measured in 12 weeks old.

**Parameters**	**NS**	**HS**
BW (g)	313.71 ± 10.70	297.00 ± 14.13
HR (bmp)	374.03 ± 12.27	389.35 ± 23.04
SBP (mmHg)	119.53 ± 8.31	172.23 ± 5.79^*^
LVID-d (mm)	5.89 ± 0.86	6.30 ± 0.77
IVS-d (mm)	1.83 ± 0.13	2.28 ± 0.14^*^
LVPW-d (mm)	1.81 ± 0.18	2.15 ± 0.17^*^
EF (%)	78.28 ± 9.63	71.86 ± 7.39
FS (%)	48.59 ± 9.36	41.34 ± 5.71
E/A	1.88 ± 0.12	3.21 ± 0.07^*^
LV mass corrected	612.05 ± 126.10	853.92 ± 117.50^*^

*Variables were expressed as means ± SD. NS, Normal group; HS, High Salt group; BW, body weight; HR, heart rate; SBP, systolic blood pressure; LVID-d, left ventricular internal dimensions at the end-diastole; IVS-d, interventricular septum thickness at the end-diastole; LVPW-d, left ventricular posterior wall thickness at the end-diastole; EF, ejection fraction; FS, fractional shortening; E/A, maximum peak blood flow velocity at early phase of diastole/maximum peak blood flow velocity at the end of diastole; LVmass corrected, left ventricular mass corrected*.

* *P < 0.05 vs. NS group*.

### Blood Pressure and Echocardiography Analyses

Blood pressure and heart rate analyses were conducted by a device named BP-2010E non-invasive rat tail sphygmomanometer (Softron, Japan). The working principle of this device is to measure the tail arterial pulse of rats, and a waveform of blood pressure can be obtained automatically. Rats were bound in a board, with a transducer attaching in 3 cm of rats tail root. The blood pressure and heart rate were obtained from the device automatically. We repeated for about 6 times to get a mean value that was used for the final analysis. The operations were performed at two periods of 12 weeks old and 20 weeks old of rats.

Echocardiography was used to assess the function of heart through a device named Vevo770 (VisualSonics, Toronto, Canada), which was equipped with a 30 Hz transducer. First, rats were anesthetized by continuous administration of 2–3% isoflurane and bound in a heating board in a supine position, with the rats hair in chest being cleaned by a razor. Then we applied a short-axis view of the M-mode ultrasound to measuring left ventricle internal dimensions at the end of diastole (LVID-d), left ventricular posterior wall thickness at end of diastole (LVPW-d), interventricular septum thickness at end of diastole (IVS-d), left ventricular mass corrected, left ventricular ejection fraction (EF%), and left ventricular fractional shortening (FS%). Furthermore, four-chamber view of the color Doppler ultrasound was used for measuring maximum peak blood flow velocity at early phase of diastole (E) and maximum peak blood flow velocity at the end of diastole (A) in mitral orifice. Rats were conducted at 12 weeks old and 20 weeks old and the final analysis adopted average values of 6 repeated detections.

### Hemodynamics Analysis

Before sacrificed, the rats were used to measure the blood flow parameters *via* carotid artery, such as left ventricular end-diastolic pressure (LVEDP), maximum left ventricular pressure decreasing rate (–dp/dtmax), and maximum left ventricular pressure increasing rate (+dp/dtmax). The device named Multichannel biological recorder (Chengdu Instrument Company, Sichuan), which connected with artery cannula and blood pressure transducer, were available for the experimental operations. First, we used 3% pentobarbital sodium (60 mg/kg) to anesthetize rats through intraperitoneal injection, with rats fixing on a heating board in a supine position. Next, the right carotid artery was isolated, which was ligated of the distal end and shut down of the proximal end of the artery. Then we made a V-shaped incision in the middle of right carotid artery. Finally, the artery cannula was inserted into the left ventricular and the blood flow parameters were collected for final analysis. Since these experimental operations, rats were sacrificed under 3% pentobarbital sodium anesthesia (60 mg/kg) and tissues were collected immediately for further experiments.

### Vascular Diastolic Function Analysis

After rats were sacrificed, the thoracic aorta was isolated immediately and used for detecting the vascular diastolic function. DMT620 (Softron, Japan) and Krebs-Henseleit solution were available in this process. We used acetylcholine (Ach) and sodium nitroprusside (SNP) to measure endothelium-dependent or endothelium-independent vasodilation of thoracic aorta. The detailed experimental procedures were in accordance with the previous study.

### Enzyme-Linked Immunosorbent Assay Analysis

The NT-ProBNP level in serum was detected through a specific ELISA kit of NT-ProBNP (MultiSciences Biotech Co., Ltd., Wuhan, China), which was in accordance to the instructions of ELISAkit.

### H and E and Masson Staining Analyses

The tissues of heart and aorta were isolated from rats and washed carefully in ice-cold saline for three times at least. One portion of heart and aorta were put into 4% formaldehyde solution separately for about 1 h and cut into 5-μm sections at various depths to conducted the procedures of H&E and Masson staining according to the instructions of kits. The “heart injury score” was evaluated by apoptosis, contraction band necrosis, neutrophilic infiltration, intramuscular bleeding, rupture, edema and ischemia (15).

### Reactive Oxygen Species Staining Analysis

The tissues of aorta were fixed on 4% formaldehyde solution and frozen in −80°C refrigerator. Then, the frozen tissues were incubated with Dihydroethidium (DHE) for 30 min at 37°C. Next, the tissues were washed out for three times with PBS. Finally, we applied a fluorescence microscope to observing the fluorescence image and the fluorescence intensity of DHE was used for final analysis. The remaining tissues of heart and aorta were stored at −80°C refrigerator and was used to further investigate the molecular mechanisms.

### Western Blotting Analysis

A specific protein extraction kit (Cat. No. P0013B, Beyotime Biotechnology, Jiangsu, China) was available to extract total proteins from rats tissues of heart and aorta. The proteins concentration was measured by BCA protein assay kit (Cat. No. P0012, Beyotime Biotechnology, Jiangsu, China). Then, we used 10% sodium dodecyl sulfate–polyacrylamide gel electrophoresis (SDS-PAGE) to divide the total proteins into various target proteins, and the target proteins were transferred to PVDF membranes (Millipore, Bedford, MA, USA) quickly. Afterwards, the PVDF membranes that contain target proteins were incubated with certain primary antibodies for more than 12 h at 4°C before incubation with certain secondary antibodies such as goat anti-rabbit IgG or goat anti-mouse IgG (ZSGB-Bio, Peking, China, 1:5000). The primary antibodies available in this study were as follows: Collagen type I antibody (1:1000), Collagen type III antibody (1:1000), MMP2 antibody (1:1000), MMP9 antibody (1:1000), TGF-β1 antibody (1:1000), p-Smad3 antibody (1:500), Smad3 antibody (1:1000), SOD-1 antibody (1:1000), p-Akt antibody (1:1000), Akt antibody (1:1000), p-eNOS antibody (1:500), eNOS antibody (1:1000), GAPDH antibody (1:1000), Tubulin antibody (1:1000). Finally, we applied an enhanced chemiluminescence (ECL) detection kit to measuring the target proteins band through an ECL scanner (Thermo Fisher Scientific). Image Lab 4.0.1 software was used in the present study to analyze the results and all target proteins measurements were repeated at least three times.

### Statistical Analysis

GraphPad Prism 7.0 software (GraphPad Software Inc., San Diego, CA, U.S.) was used for statistical analysis. The Student's *t*-test was used for the comparisons between two groups, while the one-way ANOVA for the comparisons of multiple groups. Experimental data were expressed as the means ± standard deviations (SD) (*n* = 12). *P*-values <0.05 represented statistically difference. *P*-values < 0.01 and *P*-values < 0.001 indicated statistically significant difference.

## Results

### High Salt Diet Induced Pre-clinical Diastolic Dysfunction of Heart in Rats

After 5 weeks high salt diet, we successfully constructed an animal model of PDD in rats, as evidenced by some measurement indexes (Table 1). First, systolic blood pressure (SBP) of rats in the HS group show a significant changes (*P* < 0.05) compared to the NS group, with SBP increasing apparently. Yet there were no obvious differences in heart rates (HR) and body weight (BW) between the two groups. Echocardiography analysis revealed that cardiac diastolic function was damaged by high salt diet. Despite the fact that ejection fraction (EF%) and fractional shortening (FS%) as well as left ventricle internal dimensions at the end of diastole (LVID-d) displayed few differences between the two groups, we found that left ventricular posterior wall thickness at end of diastole (LVPW-d), interventricular septum thickness at end of diastole (IVS-d), maximum peak blood flow velocity at early phase of diastole/maximum peak blood flow velocity at the end of diastole (E/A) and left ventricular mass corrected (LV mass corrected) in the HS group increased significantly (*P* < 0.05) in contrast to the NS group. These results show that cardiac diastolic function was damaged while cardiac systolic function remained normal, indicating that the animal model of PDD was constructed by high salt diet.

### High Salt Diet Further Promoted the Development of PDD and Eventually Resulted in Heart Failure With Preserved Ejection Fraction

**Figure 1:**
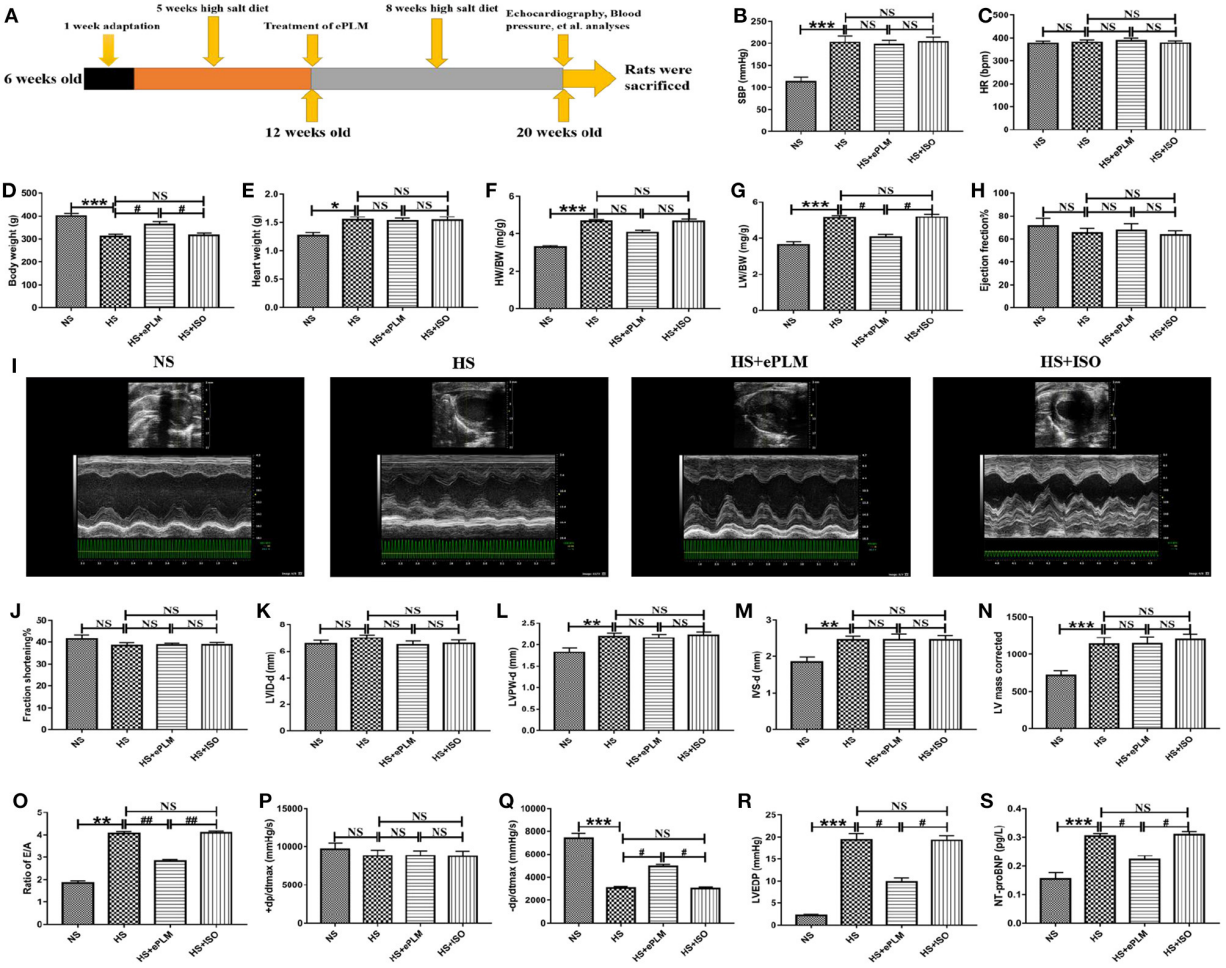
Cardiac diastolic function of HFpEF was improved by treatment of ePLM. **(A)** Overview of animal treatment protocols used in the present study (*n* = 12 each group). **(B–G)** Different parameters like SBP, HR, BW, HW, HW/BW, and LW/BW were obtained *via* many measurements to assess the general conditions of rats. **(H–O)** Parameters like EF, FS, LVID-d, LVPW-d, IVS-d, LVmass corrected and E/A ratio were investigated by echocardiography analysis. **(P–R)** Hemodynamics analysis was performed and parameters like +dp/dtmax, –dp/dtmax and LVEDP were collected. **(S)** ELISA analysis of NT-ProBNP level in serum of rats. *P < 0.05, **P < 0.01, ***P < 0.001, ^#^*P* < 0.05, and ^*##*^*P* < 0.01 means statistic significance; NS means no statistic significance. All data represent the mean ± SD of at least three experiments.

Continuously fed with high salt feed for 8 weeks, rats with 20 weeks old were evaluated through some measurement parameters. The results show that PDD further developed into HFpEF in the HS group. SBP and BW in the HS group show a significant changes (*P* < 0.001) compared to the NS group, while HR in the HS group had little differences in contrast to the NS group (Figures 1B–D). LVPW-d, IVS-d, and LV mass corrected, which were detected by the M-mode ultrasound, increased significantly (*P* < 0.01) in the HS group despite of no significant differences in LVID-d, EF%, and FS% between the two groups (Figures 1H–N). Furthermore, the color Doppler ultrasound was used to assess the value of E/A ratio, and we found a significant increasing (*P* < 0.001) of this indices in the HS group compare with the NS group (Figure 1O). In addition, we also measured the indices like left ventricular end-diastolic pressure (LVEDP), maximum left ventricular pressure decreasing rate (–dp/dtmax) and maximum left ventricular pressure increasing rate (+dp/dtmax). The results show that there was no significant differences in +dp/dtmax of the two groups, while LVEDP and –dp/dtmax displayed a obvious changes (*P* < 0.001) between the two groups, with LVEDP increasing while –dp/dtmax reducing in the HS group in contrast to the NS group (Figures 1P–R). All the data demonstrated that we successfully constructed an animal model of HFpEF by fed with high salt feed. The data also proved the fact that PDD can develop into HFpEF through high salt diet.

### Treatment of Early Passive Leg Movement Alleviated Deterioration of Cardiac Diastolic Function of HFpEF

HFpEF is a chronic disease and deteriorates gradually following aging, with cardiac diastolic dysfunction being a typical symptom. In the present study, we treated rats in 12 weeks old with ePLM to assess whether treatment of ePLM prevents against the development of PDD into HFpEF and the animal treatment protocols are summarized in Figure 1A. We obtained a positive result that treatment of ePLM exerted a protective role in deterioration of cardiac diastolic function of HFpEF. First, general parameters like SBP, HR, Heart weight (HW), and HW/BW did not see significant improvements through treatment of ePLM (Figures 1B–F). While others such as BW and Lung weight (LW)/BW acquired obvious improvements (*P* < 0.05) with treatment of ePLM (Figures 1D,G). Unfortunately, we did not find apparent improvements by treatment of ePLM according to the data collected in the M-mode ultrasound analysis. Parameters like EF%, FS%, LVID-d, LVPW-d, LV mass corrected, and IVS-d did not have apparent changes in the groups of HS group, HS+ePLM group and HS+ISO group (Figures 1H–N). But the color Doppler ultrasound and hemodynamics analyses show a amazing result. Ratio of E/A, –dp/dtmax, and LVEDP revealed significant improvements (*P* < 0.05) through treatment of ePLM despite of no obvious changes in +dp/dtmax (Figures 1O–R). In addition, we observed from the ELISA analysis that NT-proBNP level decreased significantly (*P* < 0.05) in the HS+ePLM group compared to the HS group and the HS+ISO group, suggesting that treatment of ePLM can alleviate the development of HFpEF (Figure 1S). We concluded from these data that treatment of ePLM can alleviate deterioration of cardiac diastolic function of HFpEF, but cannot reverse the cardiac remodeling, indicating that treatment of ePLM might act as a novel effective precaution approach for HFpEF.

### Treatment of Early Passive Leg Movement Alleviated Pathological Injury in Heart Tissue of HFpEF

**Figure 2:**
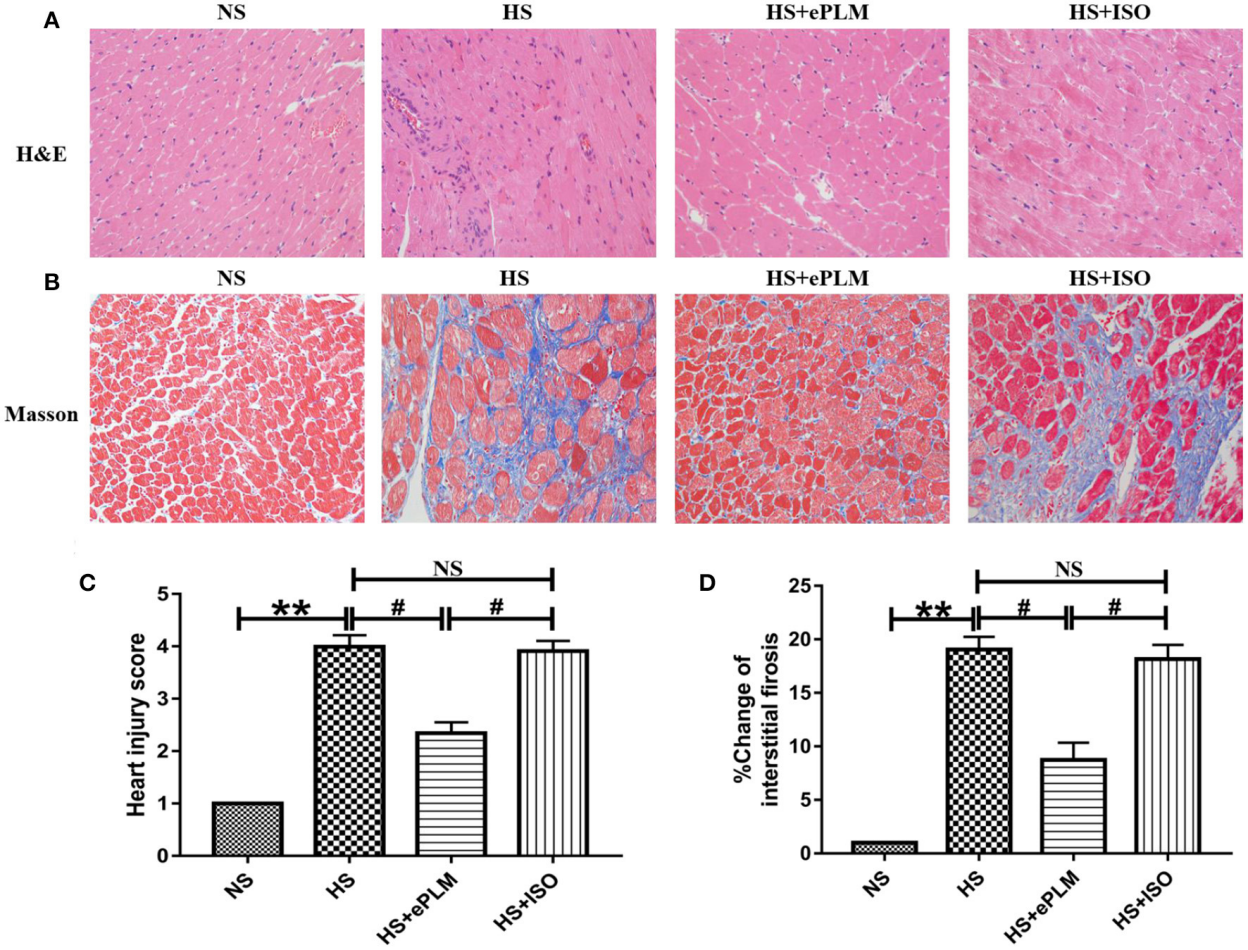
Pathological injuries in heart of rats were significantly improved by treatment of ePLM. **(A,C)** H&E staining of heart tissues and heart injury score. **(B,D)** Massion staining analysis was performed to evaluate the area of cardiac fibrosis (*n* = 12 each group). **P < 0.01 and ^#^*P* < 0.05 means statistic significance; NS means no statistic significance. All data represent the mean ± SD of at least three experiments.

Pathological injury in heart tissue is a typical feature of HFpEF. In the present study, we evaluated the pathological changes in heart tissue through H&E and Masson staining analyses. First, the result of H&E staining analysis show a significant improvement (*P* < 0.05) with treatment of ePLM in the HS+ePLM group compared with the HS group and the HS+ISO group (Figures 2A,C). Similar to the result of H&E staining analysis, the fibrotic area detected by Masson staining analysis reduced obviously (*P* < 0.05) in the HS+ePLM group in contrast to the HS group and the HS+ISO group (Figures 2B,D). The results suggested that treatment of ePLM can alleviate pathological injury in heart tissue of HFpEF, indicating treatment of ePLM being a novel non-pharmacological approach for HFpEF.

### Treatment of Early Passive Leg Movement Inhibited Cardiac Fibrosis of HFpEF

**Figure 3:**
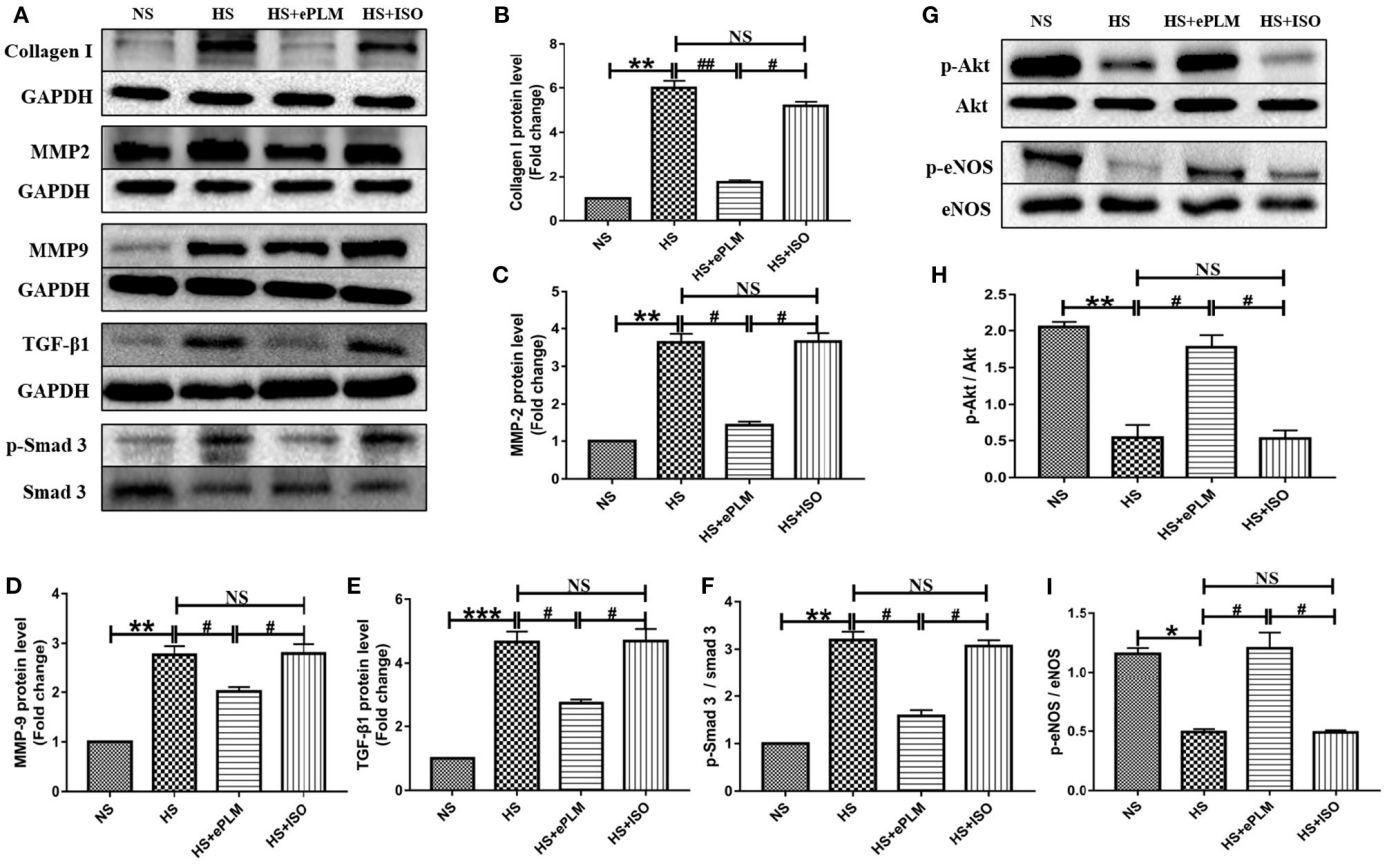
Treatment of ePLM can inhibit cardiac fibrosis *via* TGF-β1/Smad3 and Akt/eNOS signaling pathways potentially. **(A,G)** Western blotting analysis of protein levels in rats heart tissues (*n* = 12 each group). **(B–D)** Statistical analyses of protein levels (Collagen I, MMP2, and MMP9). **(E,F)** The protein levels of TGF-β1/Smad3 signaling pathway (TGF-β1 and p-Smad3). **(H,I)** The protein levels of Akt/eNOS signaling pathway (p-Akt and p-eNOS). *P < 0.05, **P < 0.01, ***P < 0.001, ^#^*P* < 0.05, and ^*##*^*P* < 0.01 means statistic significance; NS means no statistic significance. All data represent the mean ± SD of at least three experiments.

In order to explore the mechanism of treatment of ePLM in HFpEF, we conducted western blotting analysis to measure the protein level in heart tissue. First, the protein level of fibrotic factor (Collagen type I) increased significantly (*P* < 0.01) in heart tissues of the HS group and the HS+ISO group compared to the NS group, while this effect was significantly inhibited (*P* < 0.01) by treatment of ePLM in the HS+ePLM group (Figures 3A,B). The proteins of MMP2 and MMP9 exert an important role in maintaining the balance of extracellular matrix and participate in the process of fibrosis. In the present study, we observed that the protein expression levels of pro-fibrotic genes (MMP2 and MMP9) increased significantly (*P* < 0.01) in heart tissues of the HS group and the HS+ISO group compared with the NS group, while the effect was significantly inhibited (*P* < 0.05) by treatment of ePLM in the HS+ePLM group (Figures 3A–D). The changes of the protein levels (Collagen I, MMP2, and MMP9) suggested that high salt diet and ePLM had a significant influence on cardiac fibrosis, with high salt diet inducing cardiac fibrosis while treatment of ePLM inhibiting cardiac fibrosis. The TGF-β1/Smad3 signaling pathway exerts an important role in the process of fibrosis. Thus, we further investigated the protein levels of TGF-β1 and p-Smad3 by the western blotting analysis. High salt diet upregulated the expression of TGF-β1 and the ratio of p-Smad3 to total Smad3, while the effect was inhibited by treatment of ePLM (Figures 3A–F), indicating that the TGF-β1/Smad3 signaling pathway might participate in the beneficial effect of treatment of ePLM on high salt diet-induced cardiac fibrosis. Akt/eNOS signaling pathway has been reported by several studies to be associated crucially with fibrosis. Therefore, we also measured the protein levels of p-Akt and p-eNOS. The results show that the protein levels of p-Akt and p-eNOS reduced significantly (*P* < 0.01) in heart tissues of the HS group and the HS+ISO group compared to the NS group, while this effect was significantly inhibited (*P* < 0.05) by treatment of ePLM in the HS+ePLM group (Figures 3G–I), suggesting that Akt/eNOS signaling pathway might also play an important role in the process of cardiac fibrosis.

### Treatment of Early Passive Leg Movement Alleviated the Injury of Blood Vessel in HFpEF

**Figure 4:**
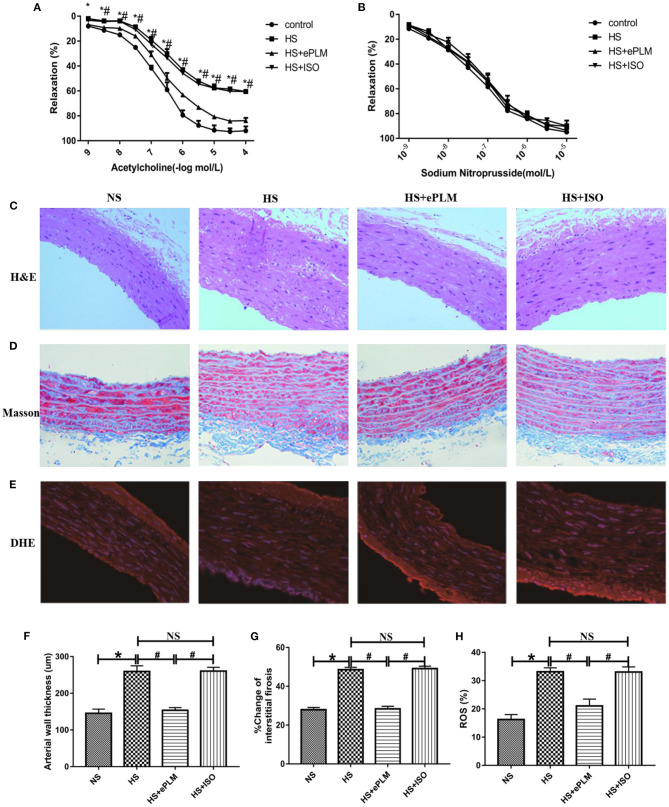
Treatment of ePLM alleviated the injury of blood vessel in HFpEF. **(A,B)** Endothelium-dependent vasodilation and endothelium-independent vasodilation were detected *via* vascular diastolic function analysis by using acetylcholine (Ach) and sodium nitroprusside (SNP) (*n* = 12 each group). **(C,F)** H&E staining of blood vessel and measurement of arterial wall thickness. **(D,G)** Massion staining analysis was conducted to evaluate the area of vascular fibrosis. **(E,H)** The level of ROS was measured by DHE staining. *P < 0.05 and ^#^*P* < 0.05 means statistic significance; NS means no statistic significance. All data represent the mean ± SD of at least three experiments.

It is well-known that the injury of blood vessel is a typical pathological change to HFpEF. Alleviating blood vessel injury can improve the prognosis of HFpEF. Thus, we also evaluated the function of blood vessel in HFpEF through vascular diastolic function analysis in the present study. Acetylcholine (Ach) was used to measure endothelium-dependent vasodilation and sodium nitroprusside (SNP) was used to measure endothelium-independent vasodilation. The results show that endothelium-dependent vasodilation was damaged seriously (*P* < 0.05) by the high salt diet in the HS group and the HS+ISO group compared to the NS group, while this effect was inhibited significantly (*P* < 0.05) through the treatment of ePLM in the HS+ePLM group (Figure 4A). However, treatment of ePLM only improved endothelium-independent vasodilation dysfunction in a certain degree (Figure 4B). In summary, treatment of ePLM improved the vascular diastolic dysfunction of HFpEF. We further detected the pathological changes of blood vessel through H&E and Masson staining analyses. H&E staining analysis show that arterial wall thickness increased significantly (*P* < 0.05) in the HS group and the HS+ISO group compared with the NS group, while this effect was inhibited significantly (*P* < 0.05) by treatment of ePLM in the HS+ePLM group (Figures 4C,F). Masson staining analysis show that the fibrotic area increased significantly (*P* < 0.05) in the HS group and the HS+ISO group compared to the NS group, while reduced significantly (*P* < 0.05) in the HS+ePLM group (Figures 4D,G). In addition, we also measured the changes of reactive oxygen species (ROS) by DHE staining, the result show that the level of ROS increased significantly (*P* < 0.05) in the HS group and the HS+ISO group in contrast to the NS group, while reduced significantly (*P* < 0.05) in the HS+ePLM group (Figures 4E,H). All these data suggested that treatment of ePLM alleviated the injury of blood vessel in HFpEF.

### Treatment of Early Passive Leg Movement Inhibited Vascular Fibrosis of HFpEF

**Figure 5:**
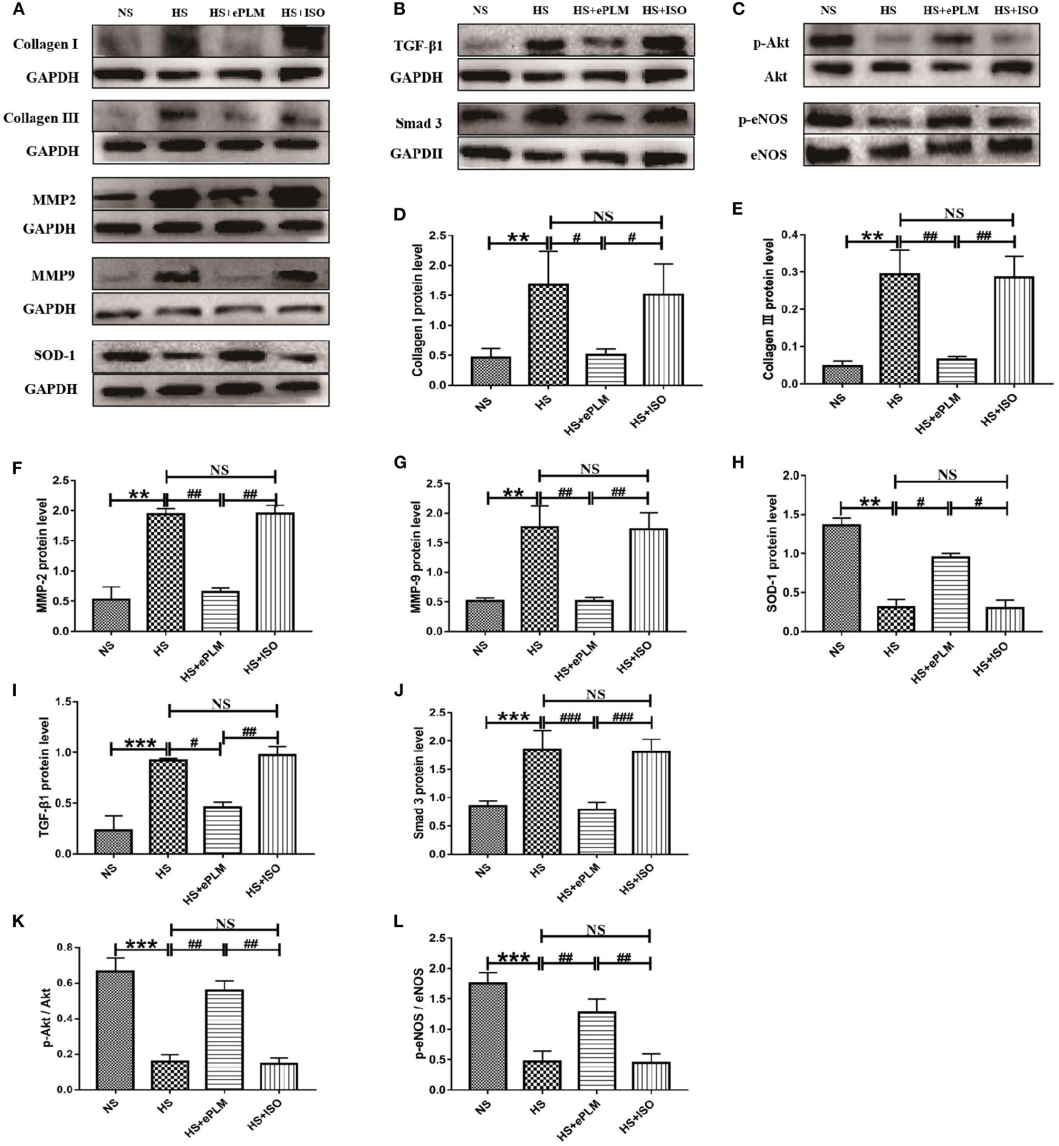
Treatment of ePLM inhibited vascular fibrosis *via* TGF-β1/Smad3 and Akt/eNOS signaling pathways potentially. **(A–C)** Western blotting analysis of protein levels in blood vessel of rats (*n* = 12 each group). **(D–G)** Statistical analyses of protein levels (Collagen I, Collagen III, MMP2, and MMP9). **(H)** The protein level of antioxidant SOD-1. **(I,J)** The protein levels of TGF-β1/Smad3 signaling pathway (TGF-β1 and Smad3). **(K,L)** The protein levels of Akt/eNOS signaling pathway (p-Akt and p-eNOS). **P < 0.01, ***P < 0.001, ^#^*P* < 0.05, ^*##*^*P* < 0.01, and ^*###*^*P* < 0.001 means statistic significance; NS means no statistic significance. All data represent the mean ± SD of at least three experiments.

Vascular fibrosis serves as an important part of HFpEF. Thus, we conducted western blotting analysis to measure the protein levels in blood vessel. The protein levels of fibrotic factors (Collagen type I and Collagen type III) increased significantly (*P* < 0.01) in blood vessel of the HS group and the HS+ISO group compared to the NS group, while this effect was significantly inhibited (*P* < 0.01) by treatment of ePLM in the HS+ePLM group (Figures 5A–E). Moreover, the protein levels of pro-fibrotic genes (MMP2 and MMP9) increased significantly (*P* < 0.01) in blood vessel of the HS group and the HS+ISO group compared with the NS group, while the effect was significantly inhibited (*P* < 0.01) by treatment of ePLM in the HS+ePLM group (Figures 5A–G). The results show that high salt diet and treatment of ePLM had a significant influence in vascular fibrosis, with high salt diet inducing vascular fibrosis while treatment of ePLM inhibiting vascular fibrosis. The protein of SOD-1 exerts a function of anti-oxidation. In this study, we found that the level of SOD-1 increased significantly (*P* < 0.05) by treatment of ePLM (Figures 5A,H). We also investigated the protein levels of TGF-β1 and Smad3 and found that high salt diet upregulated the expression of TGF-β1 and Smad3, while the effect was inhibited by treatment of ePLM (Figures 5B,I,J), indicating that the TGF-β1/Smad3 signaling pathway might participate in the protective effect of treatment of ePLM on high salt diet-induced vascular fibrosis. In addition, we measured the protein levels of p-Akt and p-eNOS and the results show that the protein levels of p-Akt and p-eNOS reduced significantly (*P* < 0.01) in blood vessel of the HS group and the HS+ISO group compared to the NS group, while this effect was significantly inhibited (*P* < 0.01) by treatment of ePLM in the HS+ePLM group (Figures 5C–L), suggesting that Akt/eNOS signaling pathway might also play an important role in the process of vascular fibrosis.

## Discussion

In the present study, we first successfully constructed an animal model of PDD in rats at 12 weeks old, and PDD eventually developed into HFpEF through high salt diet. Then we treated rats in period of PDD with early passive leg movement (ePLM) to evaluate its effect on HFpEF. The study show that treatment of ePLM exerted a protective effect on HFpEF, implicating that treatment of ePLM as a promising novel non-pharmacological approach for HFpEF.

It is reported that mitochondrial homeostasis serves as an important role in the progression of heart failure (16). Further studies concluded that overload of Ca^2+^ in mitochondrion will open the mitochondrial permeability transition pore (mPTP) and contribute to mitochondrial swelling, which will result in injury of mitochondrion, apoptosis, cardiac remodeling, and ultimately development into heart failure (17). HFpEF is a severe and chronic disease and belongs to heart failure, which results in numerous deaths of patients (6). However, there was no effective therapeutic approaches to improve the prognosis of HFpEF to date (7). Although a study recently reported that sacubitril/valsartan might ameliorate the outcome of HFpEF, but it needs more investigations to comfirm the theory (8). Thus, its urgent to explore novel therapeutic approaches for HFpEF. Several studies have reported that patients of HFpEF benefited a lot from exercising, with enhancing sport ability and improving living quality, which indicated that exercising might serve as a more promising novel non-pharmacological approach for HFpEF (13). Exercising also show a good security and effectiveness to the patients with a poor physical conditions (18). Instrumental assisted passive movement was proved as a simple and effective method of exercising (14). So our team designed and constructed a device of ePLM to pretreat rats in the period of PDD, which eventually developed into HFpEF, to evaluate the effect of treatment of ePLM on HFpEF.

Cardiac diastolic dysfunction is a typical symptom of HFpEF (2). In our study, we confirmed the theory by the findings that parameters like LVPW-d, IVS-d, LV mass corrected, E/A ratio, and LVEDP increased significantly while –dp/dtmax reduced in the HS group in contrast to the NS group. After treatment of ePLM, the condition of cardiac diastolic function was significantly improved, evidenced by the fact that ratio of E/A, –dp/dtmax, and LVEDP revealed a obvious improvement, despite of no apparent changes in LVPW-d, IVS-d, LV mass corrected, and +dp/dtmax. Consistent with the fact that NT-ProBNP serves as a strong predictive factor of heart failure events in patients with HFpEF (19), we discovered that the level of NT-ProBNP in rats serum with HFpEF decreased significantly by the treatment of ePLM. We also found that pathological injuries in heart were ameliorated after treatment of ePLM. All these data collected from the study suggested that treatment of ePLM might serve as a novel therapeutic approach for HFpEF. We further explored the molecular mechanism of treatment of ePLM through western blotting analysis. The proteins of MMP2 and MMP9 exert an important role in maintaining the balance of extracellular matrix and participate in the process of fibrosis (20). We found in this study that the protein levels of fibrotic factor (Collagen type I) and pro-fibrotic genes (MMP2 and MMP9) reduced obviously by treatment of ePLM. TGF-β1/Smad3 signaling pathway has been reported to play an important role on the development of cardiac fibrosis (21). Thus, we supposed that TGF-β1/Smad3 signaling pathway might be involved in the anti-fibrotic role of treatment of ePLM in cardiac fibrosis induced by the high salt diet. Our findings show that treatment of ePLM markedly suppressed the activation of TGF-β1/Smad3 signaling pathway during the progression of the high salt diet induced cardiac fibrosis, suggesting that this signaling pathway might be involved in the cardioprotective effects of treatment of ePLM in HFpEF. Akt/eNOS signaling pathway also has been reported to be associated crucially with fibrosis (22). Therefore, we also detected the protein levels of p-Akt and p-eNOS. The results show that the protein levels of p-Akt and p-eNOS increased significantly in heart tissues by treatment of ePLM, suggesting that Akt/eNOS signaling pathway might also play an important role in the process of cardiac fibrosis with HFpEF.

It is reported that the injury of blood vessel acts as a major part in pathological injuries of HFpEF (23). Thus, alleviating blood vessel injury might prevent against the development of HFpEF. In the present study, we observed that treatment of ePLM improved the vascular diastolic dysfunction of HFpEF through vascular diastolic function analysis. We further measured the pathological changes of blood vessel by H&E and Masson staining and discovered that pathological injuries in blood vessel of HFpEF were apparently improved by treatment of ePLM. Reactive oxygen species (ROS) are a kind of single electron reductive products that contain oxygens, which usually lead to bad effects of blood vessel like increasing vascular thickness or inhibiting release of NO (24). We detected the level of ROS by DHE staining and found that treatment of ePLM significantly reduced the level of ROS in blood vessel with HFpEF. We also measured the level of SOD-1 by western blotting analysis, with SOD-1 exerting a function of anti-oxidation (25), and observed that treatment of ePLM apparently increased the level of SOD-1 in blood vessel with HFpEF, suggesting that treatment of ePLM might balance the redox reaction to ameliorate blood vessel injury. All these data suggested that treatment of ePLM alleviated the injury of blood vessel in HFpEF. We further investigated the molecular mechanism of treatment of ePLM in blood vessel with HFpEF. The pro-fibrotic protein levels of Collagen type I, Collagen type III, MMP2, and MMP9 reduced significantly after treatment of ePLM, indicating that treatment of ePLM inhibited vascular fibrosis in HFpEF. TGF-β1/Smad3 signaling pathway and Akt/eNOS signaling pathway in blood vessel were also detected in this study and we obtained a similar result to heart tissues, suggesting that TGF-β1/Smad3 signaling pathway and Akt/eNOS signaling pathway both play an important role in the process of alleviating vascular fibrosis by treatment of ePLM in HFpEF.

However, there were some limitations in the present study. First, HFpEF is a complex chronic disease which include a series of pathological changes in tissues like lung and kidney (26). While our study only evaluated two tissues of heart and blood vessel of HFpEF, so it needs more investigations in HFpEF to further confirmed the effect of treatment of ePLM. And an *in vitro* experiment was also needed to be designed and conducted for comprehensively verifying the results.

In summary, the present study suggested that treatment of ePLM prevents against the development of HFpEF in rats. Improving cardiac and vascular injuries might be the major protective mechanisms of treatment of ePLM. Such protective abilities may derive from the inhibition of cardiac and vascular fibrosis by suppressing TGF-β1/Smad3 signaling pathway as well as activating Akt/eNOS signaling pathway. Thus, our findings implied that treatment of ePLM may serve as a more promising novel non-pharmacological approach for HFpEF.

## Data Availability Statement

The raw data supporting the conclusions of this article will be made available by the authors, without undue reservation.

## Ethics Statement

The animal study was reviewed and approved by The Animal Care and Use Committee of the Second Affiliated Hospital of Nanchang University (China).

## Author Contributions

Y-FD designed and funded the experiment. JL, X-xJ, and YF conducted the study and accomplished the paper. W-cZ, H-fJ, J-wL, and X-sC offered some assistance to the experiment. All authors contributed to the article and approved the submitted version.

## Conflict of Interest

The authors declare that the research was conducted in the absence of any commercial or financial relationships that could be construed as a potential conflict of interest.
