# Supplementary material for: Early Passive Leg Movement Prevents Against the Development of Heart Failure With Preserved Ejection Fraction in Rats
Source: Front Cardiovasc Med. 2021 Apr 21;8:655009. doi: 10.3389/fcvm.2021.655009 (PMC8096912; doi:10.3389/fcvm.2021.655009)
Supplement: Supplementary file 1 [file Data_Sheet_1.PDF]

SUPPLEMENTAL INFORMATION

Supplemental Figure 1

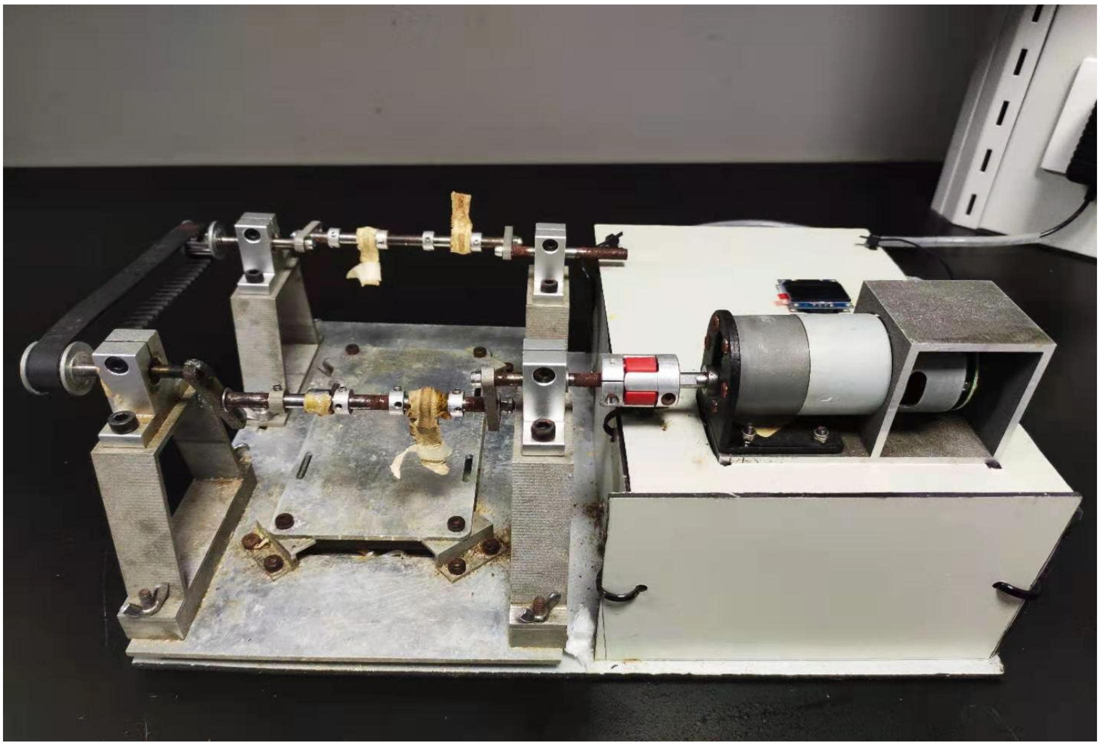

Figure S1. The device used for the pretreatment of ePLM. The working principle of this device is that the rats were anesthetized by continuous inhaling isoflurane at a supine position in the device and then turn on the switch to make an early passive leg movement (ePLM) by the device.
